# One-step fabrication of nanowire-grid polarizers using liquid-bridge-mediated nanotransfer molding

**DOI:** 10.1186/1556-276X-7-351

**Published:** 2012-06-27

**Authors:** Kyung S Park, Jeong M Dang, Myung M Sung, Soon-min Seo

**Affiliations:** 1 Department of Chemistry, Hanyang University, Seoul, 133-791, Korea; 2 Department of BioNano Technology, Gachon BioNano Research Insititute, Gachon University, Gyeonggi, 461-701, Korea

**Keywords:** Nanowire-grid polarizers, Direct printing method, Ag nanowire arrays, 81.07.Gf Fabrication nanowires, 42.79.Ci optical polarizers, 81.20.Hy, molding

## Abstract

Ag nanowire-grid polarizers (NWGPs) were prepared by a one-step fabrication method, called liquid-bridge-mediated nanotransfer molding (LB-nTM). LB-nTM is a new direct nano-patterning method based on the direct transfer of various materials from a mold to a substrate via liquid layer. We fabricated NWGPs with Ag nanowire arrays (81 nm parallel lines and 119 nm spaces) on 2.5 in. transparent substrates by LB-nTM using an Ag nanoparticle solution. The maximum and minimum transmittances of the Ag NWGP at 800 nm were 80% and 10%, respectively.

## Background

Polarizer is an indispensable device in a wide range of optical systems, including flat panel displays, microdisplays, and optical networking. Nanowire-grid polarizers (NWGPs) have been of great interest because of their excellent polarization performance and planar structure that allows them to be integrated to other thin-film optoelectronic devices. Moreover, they show good optical stability with respect to variation of the polar angle and azimuthal rotation, and excellent durability at high temperatures and under exposure to high UV flux [1]. The NWGP generally consists of fine grid of parallel metal nanowires with space and width less than the wavelength of light. For the light polarized parallel to the nanowire, it reflects, whereas for the light polarized perpendicular to the nanowire, it transmits. This type of polarizer shows very high extinction ratio between the reflected transverse electric-polarized light and the transmitted magnetic-polarized light over a wide wavelength range and incident angle.

There are several fabrication methods for generating the NWGPs, which include photolithography [2], e-beam lithography [3-6], laser interference lithography [7,8], and nanoimprint lithography [9-17]. Among these techniques, nanoimprint lithography is a cost-effective and high-throughput method for the fabrication of metal nanowire grids over a large area, but it suffers from problems. For instance, it involves additional etching or sidewall deposition processes, and continuous fabrication can be difficult because vacuum conditions are required for metal deposition. Recently, we have developed a new direct printing method for generating nanometer-scale patterns of various materials, called liquid-bridge-mediated nanotransfer molding (LB-nTM) [18]. LB-nTM is based on the direct transfer of various materials from a mold to a substrate through a liquid bridge between them. This new technique is capable of generating well-defined large-area nanowire patterns through one step and is well suited for use in automated direct printing machines. LB-nTM is the most efficient method for the fabrication of the wire-grid polarizers at low cost and low environmental impact.

**Figure 1:**
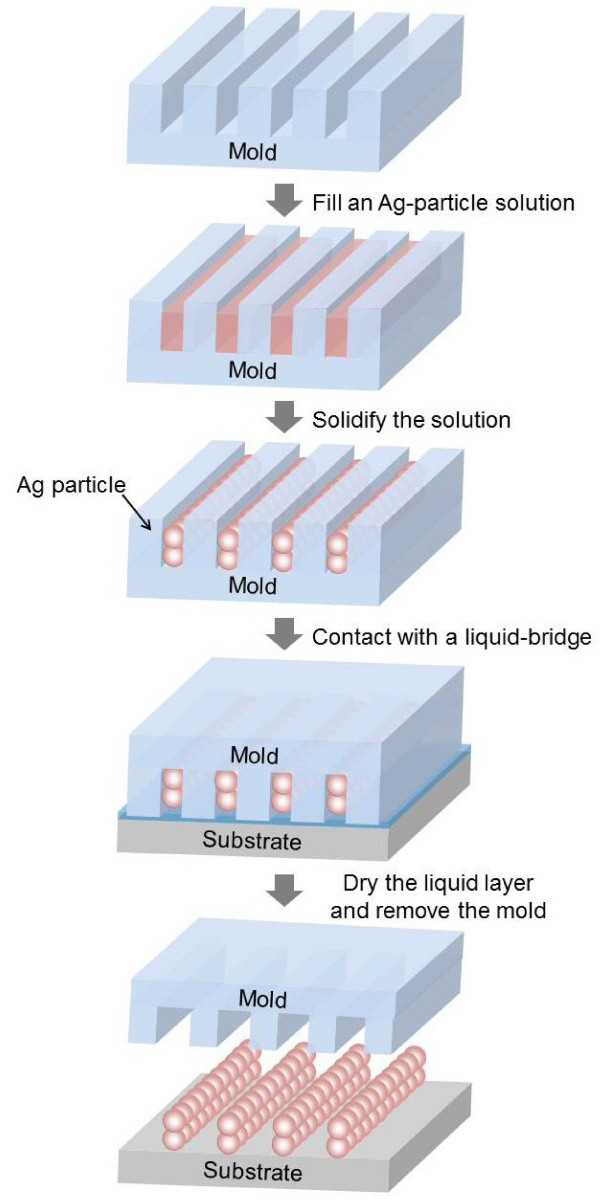
**Schematic outline.** Schematic outline of the procedure to fabricate an Ag nanowire-grid polarizer by LB-nTM.

Here, we report the one-step fabrication of the nanowire-grid polarizer using LB-nTM with an ink solution. Figure 1 illustrates the procedure to produce the Ag NWGP by LB-nTM using an Ag nanoparticle solution. First, a patterned mold fabricated by polyurethane acrylate (PUA) is filled with an Ag nanoparticle solution using selective inking [19]. The filled ink is next solidified by drying at mild temperatures (<100 °C). Almost no particles remain on the protruding surfaces of the mold as a result of the selective inking. The mold with solidified ink is then brought into contact with a substrate surface covered by a thin polar liquid layer. Due to the solidification of the ink solution, LB-nTM does not suffer from surface diffusion and can generate nanometer-scale metal lines well below 100 nm. The polar liquid layer on the substrate forms a liquid bridge (a capillary bridge) [20-23] between the substrate and the mold that contains recessed patterns. The liquid bridge allows good conformal contact between the solidified ink and the substrate. As the liquid evaporates, the attractive capillary force gradually increases, pulling the two surfaces into contact, and providing good conformal contact between them with no additional pressure to the mold. After drying, the separation of the mold from the substrate then results in the formation of the well-defined Ag nanowire grids onto the substrate.

## Methods

### Materials

Unless otherwise noted, all commercial materials were obtained from Aldrich Chemical Co. (St. Louis, MO, USA) and used without further purification. The Ag nanoparticle ink (DGP 40LT-15 C) was purchased from Advanced Nano Products (Chungcheongbuk-do, South Korea). The ink contained 20 wt.% silver nanoparticles, with a particle diameter of 40 to 50 nm, dispersed in methanol solvent. PUA (MINS-ERM, Minuta Tech. Co. LTD, Gyeonggi-do, Korea) was used to prepare the UV-curable hard molds. Polydimethylsiloxane (PDMS, Sylgard 184) was ordered from Dow Corning (Dow Corning, Midland, Michigan, USA). Deionized water was purified with a Millipore Milli Q plus system (Billerica, MA, USA), distilled over KMnO_4_, and then passed through a Millipore Simplicity system.

### Preparation of substrates

The flexible substrates employed in this study were cut from polyethylene terephthalate (PET) films (i-components Inc., Seongnam, South Korea). The PET substrates were cleaned with methanol and deionized water, and finally blow-dried with nitrogen to remove the contaminants. The Si substrates used in this research were cut from n-type (100) wafers with resistivity in the range of 1 to 5 Ω·cm. The Si substrates were initially treated by a chemical cleaning process, which involves degreasing, HNO_3_ boiling, NH_4_OH boiling (alkali treatment), HCl boiling (acid treatment), rinsing in deionized water, and blow-drying with nitrogen, proposed by Ishizaka and Shiraki, to remove contaminants [24]. A thin oxide layer was grown by placing the Si substrate in a piranha solution (4:1 mixture of H_2_SO_4_:H_2_O_2_) for 10 to 15 min. The substrate was rinsed several times in deionized water (resistivity = 18 MΩ·cm) then dried with a stream of nitrogen.

### Sample characterization

The samples were characterized by using a scanning electron microscopy (SEM, Hitachi S4800, Hitachi, Ltd., Chiyoda, Tokyo, Japan) at 15 kV and a UV–vis spectrometer (Agilent 8453 UV–vis, Agilent Technologies Inc., Santa Clara, CA, USA).

## Results and discussion

**Figure 2:**
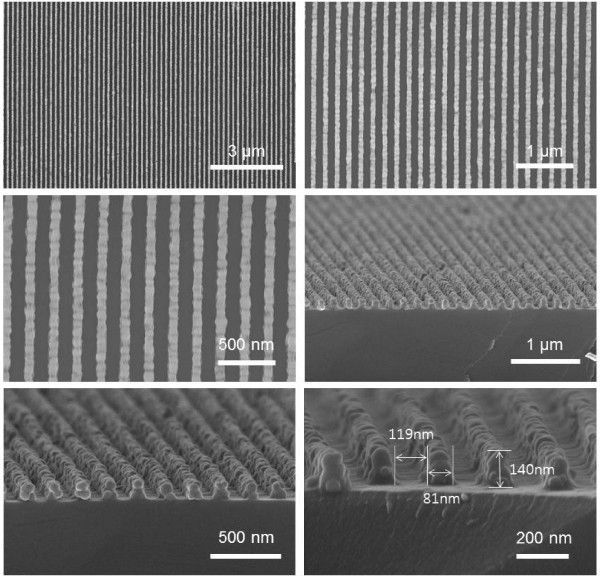
**SEM images.** SEM images of Ag nanowire patterns fabricated by LB-nTM.

For fabrication of NWGPs, Ag nanowire patterns were made on a variety of substrates such as oxidized Si(100), glass, quartz, and PET film by LB-nTM. The masters we used for fabrication of molds were silicon wafers with dense nanoscale patterns (81 nm parallel lines, 119 nm spaces, and 200 nm height), which were made by laser interference lithography and subsequent dry etchings, as described previously [25]. The molds were fabricated by casting PUA on them. After UV curing for 30 s, the PUA molds were peeled away from the masters. To pattern an array of Ag nanowires, the recessed spaces of the patterned PUA molds were filled with an Ag solution contained 20 wt.% Ag nanoparticles, with particle diameter of 40 to 50 nm, dispersed in methanol solvent. The Ag solution in the mold was solidified at 80 °C for 10 min. The mold was then placed in contact with the substrates covered by a thin ethanol layer. The substrate (1 x 1 cm^2^ area) can be uniformly covered with a 100-μm-thick ethanol layer by using 10 μl of ethanol. After the ethanol layer between the mold and the substrate dried at 70 °C for 10 min, the mold was peeled away, leaving the Ag nanowires on the substrates. Figure 2 displays SEM images of the Ag nanowire patterns made by LB-nTM. The patterns have 81 nm parallel lines, 119 nm spaces, and 140 nm heights. Compared to the dimensions of the master, the Ag patterns are only reduced in the z-direction (height) since the Ag solution locked inside the recessed spaces is solidified. The Ag patterns exhibit strong adhesion to the substrates and thus, easily passed Scotch tape adhesion tests. The patterned Ag nanowires were thermally endurable up to 500 °C but slightly shrank (about 5%) in volume.

**Figure 3:**
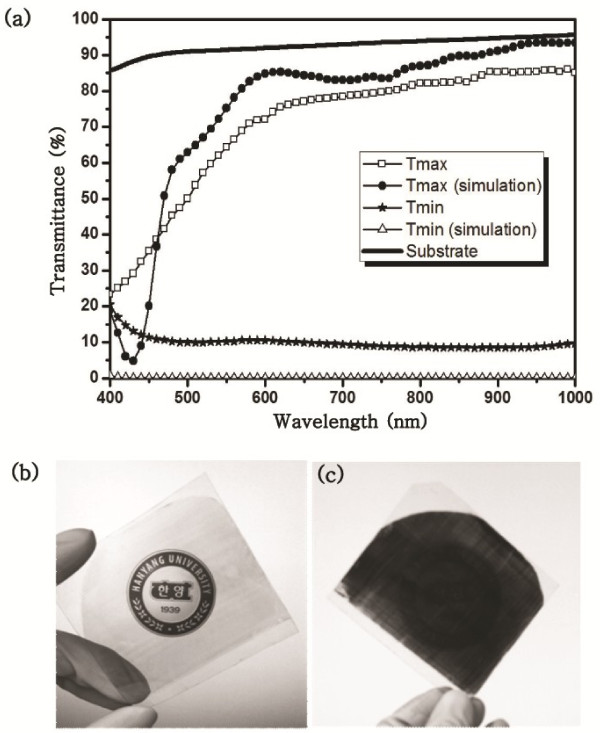
**Analysis data of Ag NWGP.** (**a**) Transmittance curves of an Ag NWGP on PET substrates. (**b**) Polarized light of an LCD panel passing through the Ag NWGP and (**c**) blocked after rotating the Ag NWGP.

We fabricated NWGPs on transparent substrates using LB-nTM. The Ag nanowire arrays with 81 nm parallel lines and 119 nm spaces were made on 2.5 in. PET substrates by LB-nTM using an Ag-nanoparticle solution. The transmittance curves of the Ag NWGP were measured from 400 to 1,000 nm using a UV/visible spectrometer in polarizing mode. The detected light showed a maximum and minimum transmittance as the NWGP was rotated. The maximum transmittance (T_max_) was observed when the Ag nanowires of the NWGP were parallel to the polarized light of the UV/visible spectrometer, and the minimum transmittance (T_min_) was observed when the nanowires were perpendicular to the light. Figure 3a shows the transmittance curves, which are similar to the theoretical expectations simulated by the Gsolver program. A little difference between the experimental and the simulated data might be due to the imperfect line width, shape, and smoothness of the Ag nanowires made by LB-nTM. The maximum and minimum transmittances of the Ag NWGP at 800 nm were 80% and 10%, respectively. The polarization of light can be confirmed simply with a liquid crystal display (LCD) monitor by rotating the NWGP, as shown in Figures 3b and c. The brightness of the monitor through the Ag NWGP was changed by rotating it. The Ag NWGP fabricated on a quartz substrate was stable up to temperatures of about 500 °C with only 1% reduced in transmittance.

## Conclusions

In summary, we described a one-step fabrication of an Ag NWGP using LB-nTM. Ag nanowire arrays, produced by LB-nTM using an Ag-particle solution, exhibited fine fidelity with a high aspect ratio (approximately 1.73). The Ag NWGPs were fabricated on 2.5 in. PET substrates and showed a high transmission and a contrast ratio for the range of visible light. This method is an ideal fabrication technique for automated direct printing machines that produce large area NWGPs on diverse substrates with no additional steps.

## Competing interests

The authors declare that they have no competing interests.

## Authors' contributions

MMS conceived and designed the experiment. KSP and JMD performed the experiment and analyzed the data. SMS contributed to materials and analysis. KSP and MMS co-wrote the paper. All authors read and approved the final manuscript.
